# From proteomic data to networks: statistics and methods reveal ciliary protein interaction landscape

**DOI:** 10.1186/2046-2530-4-S1-P90

**Published:** 2015-07-13

**Authors:** Q Lu, K Koutroumpas, K Boldt, J Van Reeuwijk, N Katsanis, F Képès, R Roepman, M Ueffing, RB Russell

**Affiliations:** 1 Cell Networks, Bioquant, University of Heidelberg, Heidelberg, Germany; 2 Institute of Systems and Synthetic Biology, Genopole, CNRS, Université d'Evry, Evry, France; 3 Division of Experimental Ophthalmology and Medical Proteome Center, Eberhard-Karls Universität Tübingen, Tübingen, Germany; 4 Department of Human Genetics and Radboud Institute for Molecular Life Sciences, Radboud University Medical Center, Nijmegen, The Netherlands; 5 Center for Human Disease Modeling, Department of Cell Biology, Duke University, Durham, NC, USA

## Objective

The assembly of protein interaction networks (PIN) is an important step to understand the biological function of proteins. Affinity purification coupled to mass spectrometry (AP-MS) has become the technique of choice for the assembly and analysis of PINs. However, most current studies, especially in human cells, are focused on specific biological systems (*e.g*. the cilium) resulting in datasets of a small to intermediate scale. In such cases, methods that developed for genome-scale datasets are of limited utility. We propose here a framework that is specifically designed for the analysis of incomplete proteomic data focused on ciliary function and ciliopathies.

## Methods

The proposed framework consists of three steps. Initially, a revised Socio-Affinity algorithm [1] is applied to quantify the pairwise protein interaction affinities. After filtering hits from noise the constructed PIN is mined for protein clusters using a novel graph-clustering algorithm. Finally, Principle Component Analysis (PCA) is used to assess the quality of detected complexes.

## Results

We applied our algorithm to data from more than 400 TAP-MS experiments, using over 200 ciliary genes as baits. Weighted PINs consisting of low, medium and high confidence interactions were extracted from the data, and for each network a set of protein complexes is reported. Several known ciliary complexes have been successfully identified, while novel ciliary complexes are predicted.

## Conclusion

We demonstrate a computational framework that can deal with context specific proteomic data. Application to experimental data focusing on cilia provides a ciliary protein interaction landscape with the ciliary biological processes/functions in the centre.

Equal contribution by Q. Lu and K. Koutroumpas
